# Identification of micro-recurrent lesions using methylene blue for mediastinal ectopic parathyroid adenoma: a case report

**DOI:** 10.1186/s44215-023-00096-5

**Published:** 2023-08-01

**Authors:** Shunsuke Nomura, Hideki Ujiie, Aki Fujiwara-Kuroda, Kichizo Kaga, Masato Aragaki, Jun Muto, Ryohei Chiba, Gaku Yamazaki, Kento Wakabayashi, Yoshihiro Matsuno, Tatsuya Kato

**Affiliations:** 1 Department of Thoracic Surgery, Hokkaido University Hospital, N14W5, Kita-Ku, Sapporo, Hokkaido 060-8648 Japan; 2 Department of Surgical Pathology, Hokkaido University Hospital, N14W5, Kita-Ku, Sapporo, Hokkaido 060-8648 Japan

**Keywords:** Mediastinal parathyroid adenoma, Recurrence, Methylene blue, Video-assisted thoracoscopic surgery

## Abstract

**Background:**

Mediastinal ectopic parathyroid adenomas are rare, and several methods are used to diagnose and localize them preoperatively. Technetium-99m methoxy isobutyl isonitrile scintigraphy has been used to diagnose parathyroid tumors. However, it is difficult to identify tumors buried in adipose tissue during surgery.

**Case presentation:**

We report a case in which methylene blue effectively identified small recurrent lesions in a mediastinal ectopic parathyroid adenoma. After intravenous injection of methylene blue prior to surgery, the stained parathyroid adenoma was easily identifiable. Additionally, we identified other small recurrent lesions that could not be confirmed on preoperative imaging using real-time in vivo imaging guidance during surgery. Using this strategy, complete macroscopic resection can be performed during video-assisted thoracic surgery.

**Conclusion:**

Preoperative intravenous methylene blue injection is useful for identifying small recurrent lesions, even in cases of mediastinal ectopic parathyroid adenoma with suspected recurrence.

## Background

Mediastinal ectopic parathyroid adenomas are rare, accounting for only 1–2% of all parathyroid adenomas [1, 2]. Several methods have been used to preoperatively diagnose and localize mediastinal ectopic parathyroid adenomas. Technetium-99m methoxy isobutyl isonitrile (99mTc-MIBI) scintigraphy is an efficient technique for diagnosing parathyroid tumors. However, identifying tumors buried in adipose tissue during surgery can be difficult [3]. In contrast, intravenous methylene blue administration has been reported to be effective in staining the parathyroid glands, and its usefulness in parathyroid localization has recently been acknowledged [4–6]. However, there is a dearth of knowledge regarding the identification of micro-recurrent lesions in patients with suspected mediastinal ectopic parathyroid adenoma recurrence.

Here, we present a case of recurrent mediastinal ectopic parathyroid adenoma in which recurrent microscopic lesions were identified using methylene blue. This case report aimed to evaluate the usefulness of preoperative intravenous injection of methylene blue for identifying recurrent microscopic lesions in cases of suspected recurrence of mediastinal ectopic parathyroid adenomas. These findings provide valuable information for the diagnosis of patients with mediastinal ectopic parathyroid adenomas and may aid in improving their surgical outcomes.

## Case presentation

An 80-year-old female patient presented with complaints of headaches and lightheadedness. She had undergone thoracoscopic surgery for a mediastinal ectopic parathyroid adenoma 15 years before her current presentation. Blood tests showed elevated parathyroid hormone (PTH) and calcium levels, which led to the suspicion of secondary hyperparathyroidism. A 99mTc-MIBI scintigraphy revealed increased mediastinal uptake, leading to a referral to our hospital for surgery.

**Fig. 1:**
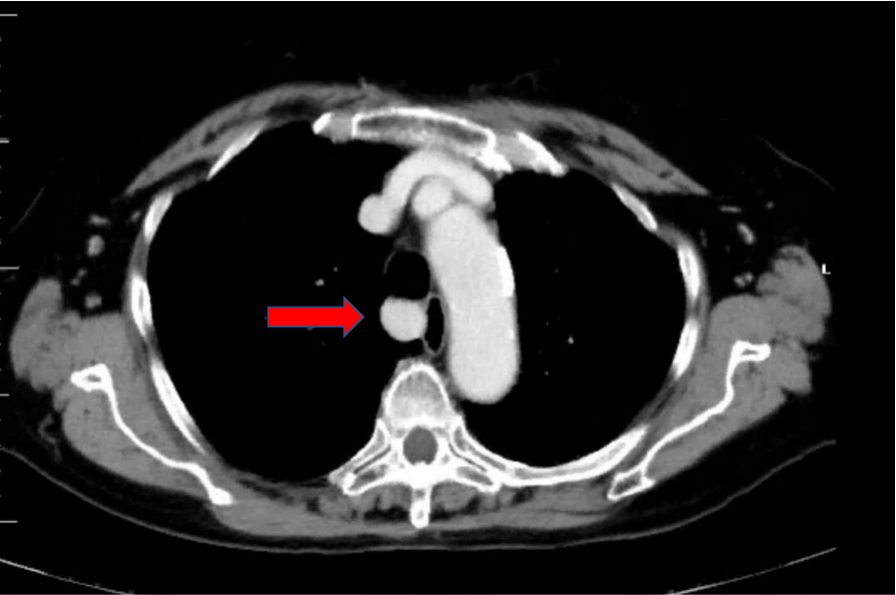
Chest computed tomography showing a small 17 × 16 mm nodule in the middle mediastinum

**Fig. 2:**
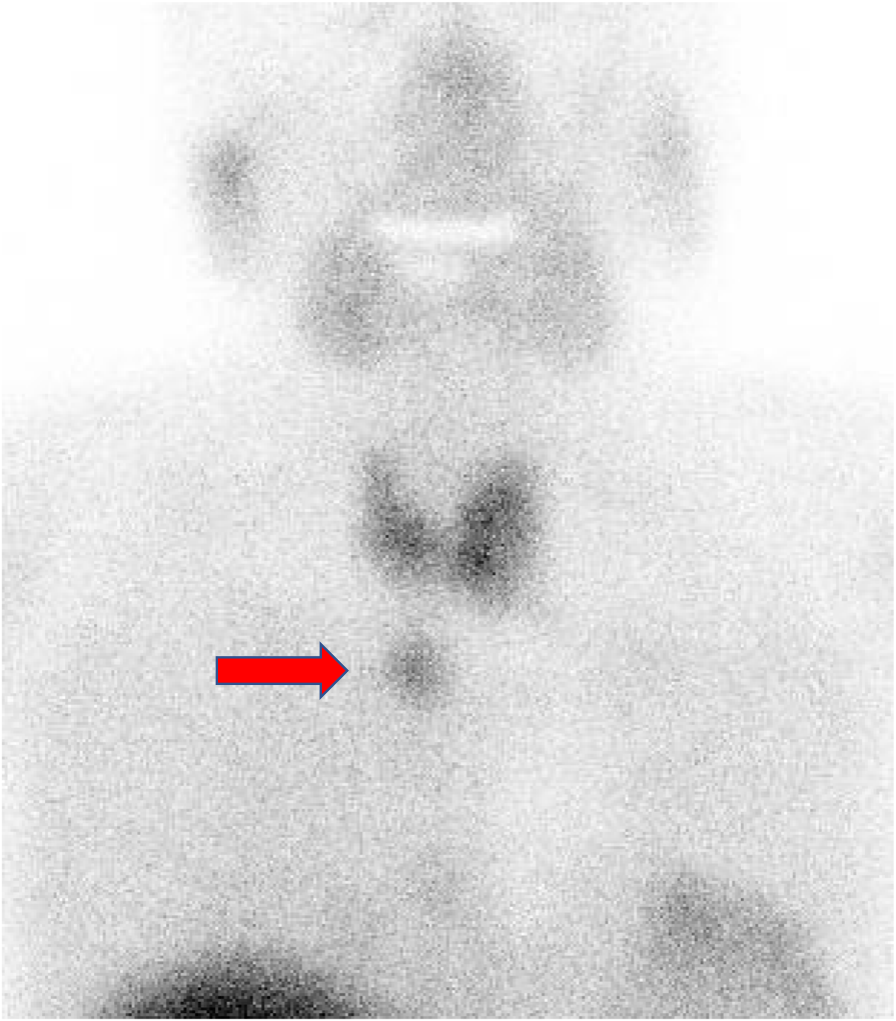
Technetiuim-99m methoxy isobutyl isonitrile scintigraphy showing an area of increased mediastinal uptake during the early phase

Upon further examination, blood tests showed a calcium level of 12.4 mg/dL (normal range, 8.8–10.1 mg/dL) and an intact PTH level of 396 pg/mL (normal range, 9.3–74.9 pg/mL). Chest computed tomography (CT) revealed a 17 × 16 mm nodule in the middle mediastinum (Fig. 1), and 99mTc-MIBI scintigraphy showed increased uptake consistent with the same site (Fig. 2). The patient was diagnosed with a recurrent mediastinal ectopic parathyroid adenoma.

**Fig. 3:**
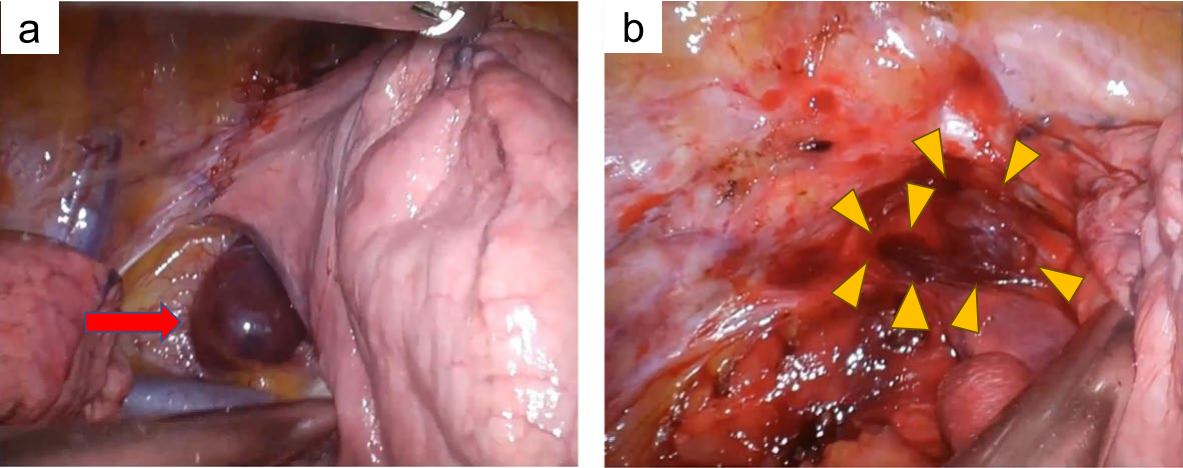
Main tumor stained with methylene blue (**a**) and two microscopic tumors stained with methylene blue on the cranial side of the tumor (**b**)

Informed consent was obtained from the patient, and consent for the use of methylene blue was also obtained. Immediately after induction of anesthesia, 75 mL of 1% methylene blue dissolved in 100 mL of saline was injected intravenously. The patient was placed in the left lateral recumbent position, and video-assisted thoracoscopic surgery (VATS) was performed. A tumor stained with methylene blue was observed and resected along with the surrounding tissue (Fig. 3a). Further exploration of the cranial side revealed two additional tumors suspected of recurrence that were resected (Fig. 3b).

The operative time was 70 min, with minimal blood loss. Histopathological findings revealed uniform round nuclei and densely packed chief cell-like cells forming nests, almost identical to those observed in a tumor excised during the initial surgery. Immunostaining was positive for PTH. The patient’s postoperative course was uneventful, and her calcium and PTH levels returned to normal.

## Discussion and conclusions

Ectopic parathyroid gland involvement frequently causes recurrent and persistent hyperparathyroidism. Ectopic parathyroid tissue is commonly found in the parapharynx, intrathyroid region, and mediastinum [7]; however, most cases are found adjacent to the superior pole of the thymus [8]. The accurate identification of mediastinal ectopic parathyroid tissue is essential for surgery. Inadequate imaging may result in longer operative times and residual or unidentifiable lesions [1].

Parathyromatosis is characterized by the emergence of functional nodules after surgery for parathyroid adenomas. Although the exact recurrence rate of parathyroid adenomas is unknown, the recurrence of hyperparathyroidism is believed to result from incomplete parathyroid tissue removal during surgery [9, 10]. During the initial surgery, the parathyroid tissue may have been overlooked, and the possibility of dissemination could not be ruled out. During the second surgery, we identified a recurrent microscopic lesion that was not detected on preoperative imaging with methylene blue. To the best of our knowledge, this is the first case report to describe the use of methylene blue to identify recurrent ectopic mediastinal parathyroid adenomas.

The method of identifying adenomas by administering methylene blue during parathyroid surgery was first reported in 1971 [4] and was subsequently introduced as a simple and safe method in several reports [5]. Methylene blue was first used to treat malaria in the 19th century. It was later used in a variety of settings, such as checking psychiatric medication status and diagnosing tuberculosis [11]. It is currently used to treat methemoglobinemia. Although the mechanism by which methylene blue is incorporated into the parathyroid gland is unknown, the parathyroid gland stains blue, thereby facilitating tumor localization and resection. Dudley reported that staining of the parathyroid gland intensifies at 1 h, persists for 20 min, and fades after 2.5 h [4]. Therefore, surgery should be initiated within 60 min of methylene blue administration. Although we could not find any reports discussing repeated use, there are reports of a half-life of 5.25–6.6 h [12], suggesting the possibility of toxicity due to volume overload if methylene blue is not detected within 2 h of administration and is administered again. Patel et al. have reported a 100% staining rate for parathyroid abnormalities [13]. Although there are no reports on the tissue depth to which parathyroid tumors stained with methylene blue can be detected, Vorst et al. reported that they were able to identify parathyroid tumors in 9 of 10 patients using near-infrared optical imaging with a low dose of intravenous methylene blue [14]. Lerchenberger et al. also compared near-infrared autofluorescence with indocyanine green imaging and reported that both techniques were effective in identifying the parathyroid glands [15]. Although not used in this case, near-infrared light may allow for better detection of parathyroid tumors.

Discoloration of the urine and skin is a common side effect of methylene blue administration, and Robert et al. reported that 120 patients who received methylene blue had no significant side effects [16]. The potential serious adverse effects include disorientation, restlessness, tachycardia, and hypertension [13]. Methylene blue is typically administered intravenously at 5–7 mg/kg. The UK National Poison Information Service recommends a dose of 4 mg/kg because of reported complications; doses exceeding 7 mg/kg can cause gastrointestinal symptoms [9] and, as a serious side effect, neurotoxicity due to serotonin toxicity [17]. The mechanism of serotonin toxicity is believed to involve the inhibition of monoamine oxidase by methylene blue. Monoamine oxidase is an enzyme involved in the breakdown of the neurotransmitter, serotonin. Simultaneous administration of selective serotonin reuptake inhibitors (SSRIs) and methylene blue may cause toxicity [18, 19]. Therefore, it is contraindicated in patients undergoing SSRIs. In the present case, 4.4 mg/kg was administered, and no serious tumor complications developed. Other precautions included an intraoperative drop in SpO_2_ [20]. This is a mechanical problem with the monitor display associated with methylene blue administration, and there are no substantial problems with blood gas analysis. In the present case, SpO_2_ could not be measured for several minutes after methylene blue administration, but the blood gas analysis was normal.

Our results suggest that preoperative intravenous injection of methylene blue can be a useful tool for identifying recurrent microscopic lesions in cases of suspected recurrence of mediastinal ectopic parathyroid adenomas. This could lead to a more accurate diagnosis and better outcomes in patients undergoing surgery for recurrent mediastinal ectopic parathyroid adenomas. Thoracoscopic surgery is an effective treatment for recurrent mediastinal ectopic parathyroid carcinoma with small recurrent lesions, which reduces operative time and minimizes invasiveness.

The present case illustrates the utility of preoperative intravenous methylene blue injection in identifying small recurrent lesions that cannot be detected on CT or 99mTc-MIBI scintigraphy in patients with suspected recurrent mediastinal ectopic parathyroid tumors. Additionally, VATS is a viable therapeutic option for the treatment of recurrent ectopic mediastinal parathyroid adenomas. These observations are informative for the diagnostic and therapeutic management of recurrent mediastinal ectopic parathyroid adenomas and may aid in optimizing surgical outcomes for these tumors.

## Data Availability

Data sharing is not applicable to this article, as no datasets were generated or analyzed during the current study.

## Abbreviations

PTH: Parathyroid hormone
CT: Computed tomography
VATS: Video-assisted thoracoscopic surgery
99mTc-MIBI: Technetium-99m methoxyisobutyl isonitrile
